# CHRONIC CONDITIONS DURING THE COVID PANDEMIC: MORTALITY PATTERNS IN THE UNITED STATES

**DOI:** 10.1093/geroni/igac059.1021

**Published:** 2022-12-20

**Authors:** Julia Kravchenko, Masudul Hoque, Igor Akushevich

**Affiliations:** Duke University School of Medicine, Durham, North Carolina, United States; Duke University, Durham, North Carolina, United States; Duke University, Durham, North Carolina, United States

## Abstract

Patients with chronic diseases, especially the older adults, are at increased risk of death during the COVID pandemic. We analyzed monthly patterns of mortality rates for patients with diabetes, arterial hypertension, cerebrovascular disease, heart failure, and kidney disease using the provisional Multiple Cause of Death data (2018-2022), for age-, gender, and race/ethnicity-specific population groups. Since population is available at annual basis, we used interpolation of population at risk to have the estimates of population at monthly basis. For all studied diseases, there were substantial increases in mortality among patients that peaked in late-Spring 2020 and Winter 2020/2021. Increases of COVID-related deaths were greater in older (aged 65+) than in younger (55-64) patients. For majority of diseases, Black patients predominantly had their maximum/peaks of COVID-related mortality in late-Spring 2020, while White, American Indian, and Hispanic patients had their max in Winter 2020/2021, and Asian patients had both peaks. Additionally to increased COVID-related deaths, higher mortality rates were also observed among patients with above diseases who did not have COVID in their death records. These increases were more pronounced in younger (55-64) than in older (65+) age groups, and they varied by the studied disease. Increased mortality not directly related to COVID could be due to the relocations of medical resources, lower access to medical care (e.g., limited use of telemedicine), non-COVID related complications caused by the earlier COVID infection, undiagnosed and/or unregistered COVID cases in patients with chronic disease, or other causes. Disease-specific differences in mortality patterns were analyzed and discussed.

